# Three-year outcome after transanal versus laparoscopic total mesorectal excision in locally advanced rectal cancer: a multicenter comparative analysis

**DOI:** 10.1186/s12885-020-07171-y

**Published:** 2020-07-20

**Authors:** F. B. de Lacy, S. X. Roodbeen, J. Ríos, J. van Laarhoven, A. Otero-Piñeiro, R. Bravo, T. Visser, R. van Poppel, S. Valverde, R. Hompes, C. Sietses, A. Castells, W. A. Bemelman, P. J. Tanis, A. M. Lacy

**Affiliations:** 1 Department of Gastrointestinal Surgery, Institute of Digestive and Metabolic Diseases, Hospital Clinic, IDIBAPS, Centro de Investigación Biomédica en Red en Enfermedades Hepáticas y Digestivas (CIBERehd), University of Barcelona, Centro Esther Koplowitz, and Cellex Biomedical Research Center, Barcelona, Catalonia Spain; 2 Department of Surgery, Amsterdam University Medical Centers, University of Amsterdam, Cancer Center Amsterdam, Meibergdreef 9, Amsterdam, The Netherlands; 3 Medical Statistics Core Facility, August Pi and Sunyer Biomedical Research Institute (IDIBAPS); Biostatistics Unit, Faculty of Medicine, Universitat Autònoma de Barcelona, Villarroel 170, 08036 Barcelona, Catalonia Spain; 4 Department of General Surgery, Jeroen Bosch Ziekenhuis, ‘s Hertogenbosch, The Netherlands; 5 Department of Surgery, Gelderse Vallei Hospital, Ede, The Netherlands; 6 Department of Gastroenterology, Institute of Digestive and Metabolic Diseases, Hospital Clinic, IDIBAPS, Centro de Investigación Biomédica en Red en Enfermedades Hepáticas y Digestivas (CIBERehd), University of Barcelona, Barcelona, Catalonia Spain

**Keywords:** Rectal cancer, Total mesorectal excision, Locoregional recurrence, TaTME

## Abstract

**Background:**

For patients with mid and distal rectal cancer, robust evidence on long-term outcome and causal treatment effects of transanal total mesorectal excision (TaTME) is lacking. This multicentre retrospective cohort study aimed to assess whether TaTME reduces locoregional recurrence rate compared to laparoscopic total mesorectal excision (LapTME).

**Methods:**

Consecutive patients with rectal cancer within 12 cm from the anal verge and clinical stage II-III were selected from three institutional databases. Outcome after TaTME (Nov 2011 - Feb 2018) was compared to a historical cohort of patients treated with LapTME (Jan 2000 - Feb 2018) using the inverse probability of treatment weights method. The primary endpoint was three-year locoregional recurrence.

**Results:**

A total of 710 patients were analysed, 344 in the TaTME group and 366 in the LapTME group. At 3 years, cumulative locoregional recurrence rates were 3.6% (95% CI, 1.1–6.1) in the TaTME group and 9.6% (95% CI, 6.5–12.7) in the LapTME group (HR = 0.4; 95% CI, 0.23–0.69; *p* = 0.001). Three-year cumulative disease-free survival rates were 74.3% (95% CI, 68.8–79.8) and 68.6% (95% CI, 63.7–73.5) (HR = 0.82; 95% CI, 0.65–1.02; *p* = 0.078) and three-year overall survival 87.2% (95% CI, 82.7–91.7) and 82.2% (95% CI, 78.0–86.2) (HR = 0.74; 95% CI, 0.53–1.03; *p* = 0.077), respectively. In patients who underwent sphincter preservation procedures, TaTME was associated with a significantly better disease-free survival (HR = 0.78; 95% CI, 0.62–0.98; *p* = 0.033).

**Conclusions:**

These findings suggest that TaTME may improve locoregional recurrence and disease-free survival rates among patients with mid and distal locally advanced rectal cancer.

## Background

Each year, 125,000 new cases of rectal cancer are diagnosed in the European Union [1] and mortality ranges between 4 and 10/100,000 per year. The therapeutic approach to rectal cancer is eminently multidisciplinary, but surgery remains the main cornerstone for cure. For mid- and low rectal tumours, total mesorectal excision (TME) along embryological-specific planes is the standard surgical treatment [2]. An intact specimen based on the principles of TME grading, the circumferential resection margin, and the distal resection margin have become the most critical factors in predicting the risk of locoregional recurrence and long-term survival [3–5].

Primary rectal cancer surgery can be performed through an open, laparoscopic, robotic, or transanal approach. The oncological superiority of one approach over the other is still a topic of debate. A recent meta-analysis showed that the risk of a suboptimal TME specimen is higher with laparoscopy compared to open surgery [6]. The transanal TME (TaTME) was developed to improve patient outcomes and the quality of the dissection, which is believed to be especially significant in mid- and low rectal tumours. Studies published to date have reported mesorectal excision completeness rates as high as 92.5 to 96%, and an even significantly higher rate of complete and near-complete mesorectal excisions compared to conventional laparoscopic TME (LapTME) [7–9]. These clinical findings suggest that TaTME is a highly promising technique, although the translation of this data into improved mid- and long-term oncological outcomes has yet to be proven. Unexpectedly, a recent study reported a pattern of rapid and multifocal locoregional recurrence after TaTME [10]. Therefore, this multicenter international study was designed with the goal of comparing the three-year oncological outcome of patients with primary locally advanced rectal cancer treated with TaTME and LapTME in three high-volume rectal cancer centers.

## Methods

### Study population

Data were obtained from one Spanish center, the Hospital Clinic of Barcelona, and two Dutch centers, the Gelderse Vallei Hospital and the Amsterdam UMC at AMC. LapTME was first introduced at the Hospital Clinic in 1994. In November 2011, TaTME became the standard of care for all patients presenting with rectal cancer that did not require abdominoperineal resection or pelvic exenteration. In February 2017, the transanal approach became standard for patients requiring an abdominoperineal resection. Gelderse Vallei Hospital is a high-volume rectal cancer institution in which TaTME was first used in 2012. At the Amsterdam UMC TaTME became the standard procedure for patients presenting with mid- and low rectal cancer from 2014 onwards. All patients with histologically proven rectal adenocarcinoma treated by TaTME were prospectively registered in a local standardised database or in the International TaTME Registry [11]. Consequently, a multicenter database was created, which included the TaTME cohort and a cohort of patients treated by LapTME between January 2000 and February 2018, through a retrospective analysis of clinical records. All three hospitals used TaTME as a standard procedure for patients with mid- and low rectal cancer until their recent participation in the COLOR III trial, a randomised study in which participants are allocated to either TaTME or LapTME [12].

For this analysis, adult patients with a solitary locally advanced rectal adenocarcinoma, according to the ACOSOG Z6051 definition (cT3/cT4, or cN1/cN2 with any cT) detected by magnetic resonance imaging (MRI) with or without transrectal ultrasonography, within 12 cm of the anal verge treated with TaTME or LapTME were included [13]. The exclusion criteria were: patients with cTisN0 or cT1-2 N0; pelvic malignancy within 5 years; severe, incapacitating disease, i.e. American Society of Anaesthesiologists (ASA) classification IV-V; procedures performed in an emergency setting; tumours previously treated by local excision; unknown cT or cM; metastatic tumours (M1); synchronous tumours; active Crohn’s or ulcerative colitis; familial risk-colorectal cancer syndromes; and patients with 30-day mortality when it was judged to have occurred as a direct result of a major active postoperative complication, which is not of primary interest. The Institutional Ethics Committees (Comité de Ética de la Investigación con Medicamentos, Beoordlingscommissie Wetenschappelijk Onderzoek, and Medisch Ethische Toetsings Commissie AMC) approved the TaTME and LapTME techniques years prior to this study in the three institutions noted, and the current study protocol was assessed and accepted by the local Institutional Review Boards. Patients provided written informed consent.

### Endpoints

The primary endpoint was three-year locoregional recurrence. Secondary endpoints included systemic recurrence, disease-free survival, and overall survival.

### Procedures and definitions

The specific staging, classification methods, and surgical procedures have been described in more detail previously [14–16]. Tumours were considered high if the distal border of the tumour was > 10 cm from the anal verge, mid if it was between 5 and 10 cm, and low in case of a distal border < 5 cm. Patients were eligible for neoadjuvant therapy in cases of cT3b-d/cT4 or cN-positive tumours below the peritoneal reflection, or if the circumferential resection margin was threatened or involved, although other factors such as extramural venous invasion were also taken into account and discussed by a multidisciplinary team. The indication to receive radiotherapy alone or in combination with chemotherapy was given depending on the institution-specific protocols. Short-course one-week radiotherapy was administered by 25 Gy in five daily fractions. Neoadjuvant long-course chemoradiotherapy was administered by continuous 5-fluorouracil (5-FU) infusion (225 mg/m^2^ for 5 days per week) or capecitabine (825 mg/m^2^ twice daily for 5 days per week), and a total dosage of 45 Gy, by a weekly dose of 9 Gy divided in 5 days each week, for a total of 5 weeks. The interval between completion of long-course chemoradiotherapy and surgery was 5 to 7 weeks at the beginning of the LapTME cohort recruitment, and, in accordance with current guidelines, was subsequently extended up to 12 weeks and associated with appropriate restaging [1].

The mesorectal specimen was analysed on the basis of four major pathological factors: the integrity of the mesorectum, graded following the Quirke method: complete, near-complete, or incomplete [5]; the circumferential resection margin, considered to be positive when the distance between the deepest portion of the tumour and the resection margin was ≤1 mm, or in the case of a positive lymph node at ≤1 mm of the radical dissection plane; the distal resection margin, considered to be positive if tumour cells were present ≤1 mm from the lower border of the tumour to the cut edge of the specimen; and the number of lymph nodes harvested. Pathological tumour response to neoadjuvant therapy was scored by the Ryan tumour regression grade (three-point TRG): TRG 1, no viable cancer cells, or single cells or small groups of cancer cells; TRG 2, residual cancer outgrown by fibrosis; and TRG 3, significant fibrosis outgrown by cancer, or no fibrosis with extensive residual cancer [17].

### Follow-up

Until follow-up was completed after 5 years, patients visited every 3 to 6 months during the first 2 years and every 6 to 12 months during the remaining 3 years. Visits included a history, physical evaluation with digital rectal examination, and determination of the carcinoembryonic antigen level. In Barcelona, imaging studies with thoracic and abdominopelvic CT scans were requested every 6 months during the first 2 years and annually during the remaining 3 years. In both Gelderse Vallei and Amsterdam UMC, imaging study was based on liver ultrasound, and CT scan was performed in case of suspicion of local recurrence or distant metastasis. Pelvic MRI and/or transrectal ultrasound-guided needle biopsy were requested when pelvic recurrence was suspected. Locoregional recurrence was defined as any recurrence in the pelvic area and had to be confirmed at least on imaging.

### Statistical analysis

Qualitative variables were expressed as absolute frequencies and percentages. Quantitative variables were reported as means or medians with their 95% confidence intervals (CI), except for follow-up periods, which were expressed as median with range. To allow for an unbiased comparison, an inverse probability of treatment weights approach was used [18]. A logistic regression model was applied, including demographic and clinical preoperative variables such as hospital, age, gender, ASA classification, body mass index (BMI), the distance of the tumour from the anal verge, cT and cN stage, and baseline threatened or involved circumferential resection margin. As the goal was to develop a balanced population that was independent of the outcome assessment, postoperative variables were not included, except for relevant variables that were assessable only after surgery (i.e., pT and pN stage, and pathological response to neoadjuvant therapy). The covariate radiologic extramural venous invasion was excluded from the model due to a significant amount of missing data because this information was not routinely reported until recent years. The covariate type of surgery (categorised as sphincter-saving or abdominoperineal resection) could not be included in the propensity score calculation due to the small number of abdominoperineal resections in the TaTME group.

A well-balanced distribution of the covariates in the weighted sample was confirmed by means standardised differences meeting a standard objective of ±0.10 [19]. The only exception was the covariate hospital, in which a standardised difference of 0.12 was achieved. Since some authors consider the cut-off point for standardised differences to be ±0.20, and given the extensive homogeneity of the rest of covariates (with standardised differences of less than ±0.02), this was finally accepted [20].

The estimation of the survival functions was carried out using the Kaplan-Meier method. The estimation of the effect of surgical procedure was performed using Cox hazard models weighted by the inverse probability of treatment weight adjusting by preservation of the sphincter (sphincter-saving surgery or abdominoperineal resection) with a cut-off at 3 years. Additional analyses using Accelerated Failure Time models were used for the analysis of time to event data in order to estimate the time ratio (TR) for the effect of the surgical procedure on acceleration in the time to the event [21].

Statistical tests were two-sided with a 5% type I error. All the analyses were carried out using SPSS version 25 (IBM) or SAS version 9.4 (SAS Institute, Inc., Cary, North Carolina).

## Results

**Fig. 1:**
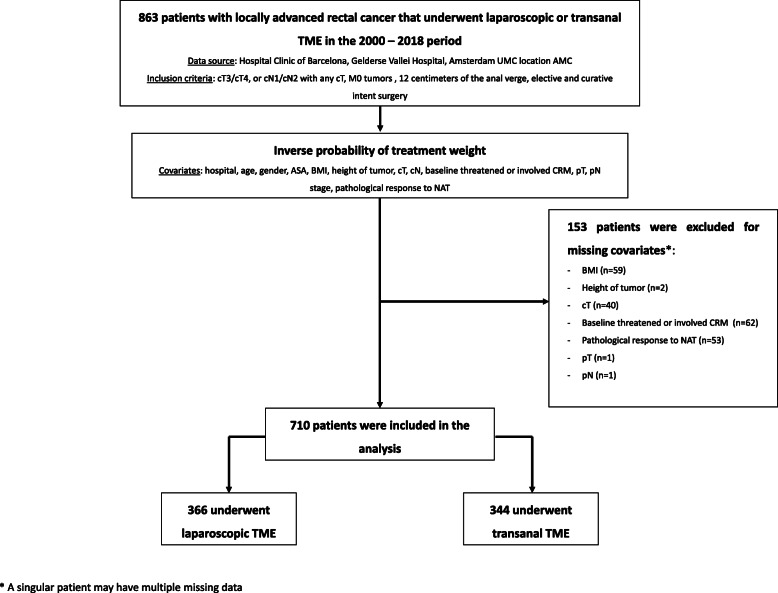
Flow diagram of study population. Abbreviations: TME, total mesorectal excision; AMC, Amsterdam University Medical Centers; ASA, American Society of Anesthesiologists; BMI, body mass index; CRM, circumferential resection margin; NAT, neoadjuvant therapy; LapTME, laparoscopic total mesorectal excision; TaTME, transanal total mesorectal excision

Between 2000 and 2018, 863 patients with primary locally advanced rectal adenocarcinoma met the inclusion criteria and were eligible. Of these, 153 (17.7%) were excluded because data were missing in the covariates selected for the inverse probability of treatment weight. This occurred mainly at the beginning of the LapTME cohort, because either systematization in reporting all the information was scarce, or the necessary investigations to obtain that data were not always performed in that period. Therefore, the final study population consisted of 710 patients, of whom 344 (48.5%) underwent TaTME and 366 (51.5%) LapTME (Fig. 1). The median follow-up in the TaTME and LapTME cohorts was 28.4 (range 0.1–83.6) and 61.1 (range 1.1–205.7) months, respectively. After truncation at 3 years, more than 30% of the patients remained at risk in both groups. Table 1 shows the selected covariates of patients treated with TaTME or LapTME, with standardised differences before and after the inverse probability of treatment weight.

**Table 1:** Selected covariates of patients treated with laparoscopic or transanal total mesorectal excision for rectal cancer, with standardised differences before and after inverse probability of treatment weighting

Variable	Surgical approach		Standardised differences	
	TaTME, No. (%)	LapTME, No. (%)	Before IPTW	After IPTW
**Total, No.**	344	366		
**Center**				
**Barcelona**	194 (56.4)	212 (57.9)	0.1189	0.1238
**Gelderse Vallei**	90 (26.1)	79 (21.5)		
**AMC**	60 (17.4)	75 (20.4)		
**Age, years (mean, 95% CI)**	66.5 (65.2–67.7)	66.4 (65.2–67.6)	0.0071	< 0.0001
**Gender**				
**Female**	104 (30.2)	137 (37.4)	0.1526	−0.007
**Male**	240 (69.7)	229 (62.5)		
**ASA**				
**I**	44 (12.7)	68 (18.5)	0.0682	0.0002
**II**	239 (69.4)	227 (62.0)		
**III**	61 (17.7)	71 (19.4)		
**BMI, kg/m**^**2**^**(mean, 95% CI)**	25.5 (25.1–25.9)	26.4 (25.7–27.0)	−0.161	−0.017
**Distance from AV, cm (mean, 95% CI)**	7.2 (6.9–7.5)	6.5 (6.1–6.8)	0.2487	0.0024
**Clinical T-stage**				
**cT1**	0 (0.0)	4 (1.1)	0.0726	0.0047
**cT2**	27 (7.8)	41 (11.8)		
**cT3**	289 (84.0)	266 (76.6)		
**cT4**	28 (8.1)	35 (10.0)		
**Clinical N-stage**				
**cN0**	148 (43.0)	113 (30.8)	−0.288	0.0005
**cN1**	155 (45.0)	181 (49.4)		
**cN2**	41 (11.9)	72 (19.6)		
**Baseline threatened/involved CRM**	94 (27.3)	101 (27.6)	−0.006	−0.009
**Pathologic response to NAT**^**a**^				
**TRG 1**	93 (38.2)	103 (36.5)	−0.144	−0.003
**TRG 2**	76 (31.2)	85 (30.1)		
**TRG 3**	74 (30.4)	94 (33.3)		
**Pathological T-stage**			−0.144	−0.003
**pT0**	40 (11.6)	35 (9.5)	−0.144	−0.003
**pTis**	1 (0.2)	0 (0.0)		
**pT1**	26 (7.5)	20 (5.4)		
**pT2**	85 (24.7)	95 (25.9)		
**pT3**	179 (52.0)	179 (48.9)		
**pT4**	13 (3.7)	37 (10.1)		
**Pathological N-stage**				
**pN0**	251 (72.9)	243 (66.3)	−0.144	−0.003
**pN1**	57 (16.5)	76 (20.7)		
**pN2**	33 (9.5)	47 (12.8)		
**Variable**	**Surgical approach**		**Standardised differences**	
	**TaTME, No. (%)**	**LapTME, No. (%)**	**Before IPTW**	**After IPTW**
**pN3**	0 (0.0)	0 (0.0)		
**pN1c**	3 (0.8)	0 (0.0)		
**Sphincter saving surgery**^**b**^	334 (97.0)	272 (74.3)	NA	NA

Abbreviations: TaTME, transanal total mesorectal excision; LapTME, laparoscopic total mesorectal excision; IPTW, inverse probability of treatment weighting; AMC, Amsterdam University Medical Centers; ASA, American Society of Anesthesiologists; BMI, body mass index; AV, anal verge; CRM, circumferential resection margin; NAT, neoadjuvant therapy; TRG, tumour regression grade; NA, not applicable

^a^ Including only patients treated with NAT. The TRG system developed by Ryan et al. was used^17^

^b^ Not included in the IPTW calculation due to the large differences between groups. It was used as an adjustment cofactor in Cox models

In both groups, similar rates of neoadjuvant therapy administration were observed (71.2% vs. 77.0%; *p* = 0.086). Short-course radiotherapy was given to 59 (24.5%) patients in the TaTME cohort and to 79 (28.1%) patients in the LapTME cohort (*p* = 0.408), while most patients in both groups received long-course chemoradiotherapy: 180 (52.3%) vs. 203 (55.4%) (*p* = 0.371). TaTME was associated with a significant reduction in abdominoperineal resection rates: 2.9% vs. 25.6%, *p* < 0.001. Similar rates of 30-day postoperative complications were observed (31.9% vs. 35.2%; *p* = 0.382).

## Histopathological outcomes and adjuvant therapy

Despite an absence of significant differences in the original multi-category mesorectal specimen variable, a significant higher rate of complete or near-complete was observed in the TaTME cohort: 98.5% vs. 93.5%, *p* = 0.0003. The rate of circumferential resection margin involvement and incidence of intra-operative rectal perforation were also lower in the TaTME group (Table 2). These findings translated into an overall better composite endpoint of poor pathological outcome for TaTME.

**Table 2:** Pathologic and adjuvant therapy outcomes after inverse probability of treatment weighting

Variable	Surgical approach		***P*** Value
	TaTME, No. (%)	LapTME, No. (%)	
**Total, No.**	344	366	
**AJCC pathological stage**			
**0**	38 (11.0)	32 (8.7)	0.8616
**I**	90 (26.1)	92 (25.1)	
**II**	123 (35.7)	119 (32.5)	
**III**	93 (27.0)	123 (33.6)	
**IV**	0 (0.0)	0 (0.0)	
**Mesorectal specimen**			
**Complete**	318 (93.2)	242 (89.3)	0.1678
**Near-complete**	20 (5.8)	13 (4.8)	
**Incomplete**	3 (0.8)	16 (5.9)	
**Distance to CRM, mm (median, 95% CI)**	10.0 (10.0–12.0)	7.5 (6.0–10.0)	0.0131
**CRM involvement**	32 (9.5)	56 (16.2)	0.0038
**Distance to DRM, mm (median, 95% CI)**	20.0 (20.0–25.0)	19.5 (15.0–20.0)	0.248
**DRM involvement**	6 (1.8)	7 (2.0)	0.6135
**Rectal perforation**	2 (0.8)	8 (3.2)	0.0262
**Composite poor pathological outcome**^**a**^	35 (10.6)	69 (24.7)	< 0.001
**Perineural invasion**	44 (13.0)	47 (18.3)	0.0109
**Lymphovascular invasion**	68 (21.4)	44 (17.0)	0.0182
**Budding**			
**no**	155 (82.8)	38 (52.7)	0.0002
**low**	23 (12.3)	32 (44.4)	
**moderate**	2 (1.0)	0 (0.0)	
**high**	7 (3.7)	2 (2.7)	
**Differential grade**			0.5589
**good**	20 (6.3)	15 (4.8)	0.5589
**moderate**	254 (80.3)	240 (77.4)	
**poor**	17 (5.3)	22 (7.1)	
**Number of lymph node harvested (median, 95% CI)**	15.0 (15.0–16.0)	14.0 (14.0–15.0)	0.0133
**Adjuvant chemotherapy**	42 (12.2)	61 (17.1)	0.0508
**Adjuvant radiotherapy**	4 (1.1)	15 (4.2)	0.0002

Abbreviations: TaTME, transanal total mesorectal excision; LapTME, laparoscopic total mesorectal excision; AJCC, American Joint Committee on Cancer; CRM, circumferential resection margin; DRM, distal resection margin

^a^ Complete or near-complete TME, and negative CRM and DRM

### Survival and recurrence analyses

**Fig. 2:**
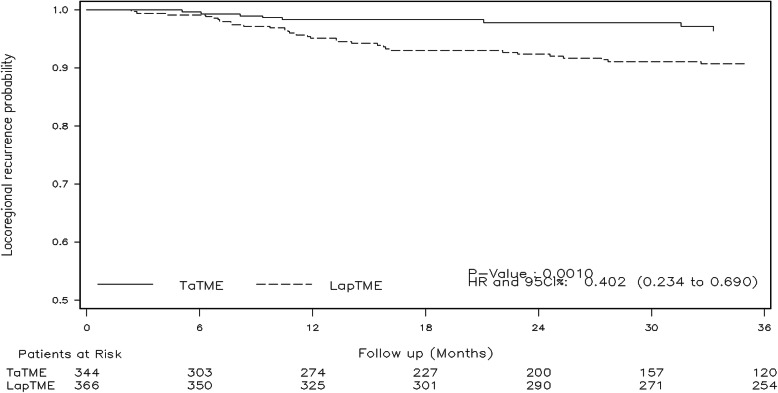
Three-year locoregional recurrence between TaTME and LapTME in patients with locally advanced rectal cancer. Abbreviations: TaTME, transanal total mesorectal excision; LapTME, laparoscopic total mesorectal excision; HR, hazard ratio; CI, confidence interval

Three years after surgery, the rates of locoregional recurrence were 3.6% in the TaTME group and 9.6% in the LapTME group Hazard Ratio (HR) = 0.4 (95% CI, 0.23–0.69; *p* = 0.001) (Fig. 2). After stratifying for sphincter-saving surgery or abdominoperineal resection, a lower rate of locoregional recurrence was maintained in those patients who underwent TaTME with sphincter preservation HR = 0.42 (95% CI, 0.24–0.73; *p* = 0.002). No difference was observed in patients with low rectal cancer HR = 0.9 (95% CI, 0.28–2.93; *p* = 0.866). In patients with cancer of the mid rectum, the rates of locoregional recurrence were 5.3% in de TaTME group and 12.3% in the LapTME group HR = 0.39 (95% CI, 0.2–0.76; *p* = 0.006). Systemic metastases were reported in 16.4% of the patients in the TaTME group and in 19.8% of the patients in the LapTME group HR = 0.93 (95% CI, 0.7–1.24; *p* = 0.615).

**Fig. 3:**
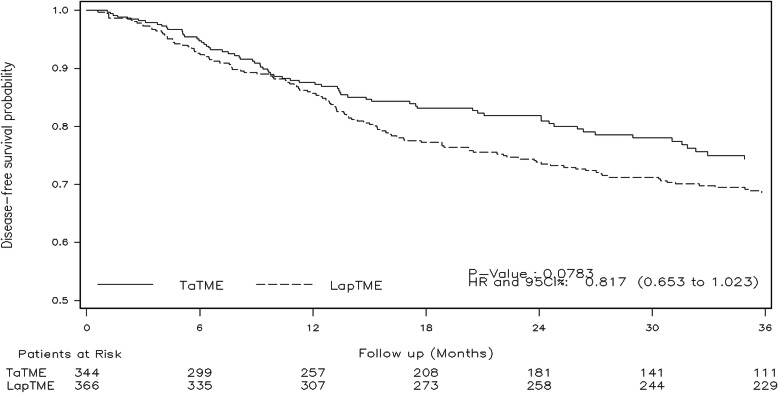
Three-year disease-free survival between TaTME and LapTME in patients with locally advanced rectal cancer. Abbreviations: TaTME, transanal total mesorectal excision; LapTME, laparoscopic total mesorectal excision; HR, hazard ratio; CI, confidence interval

**Fig. 4:**
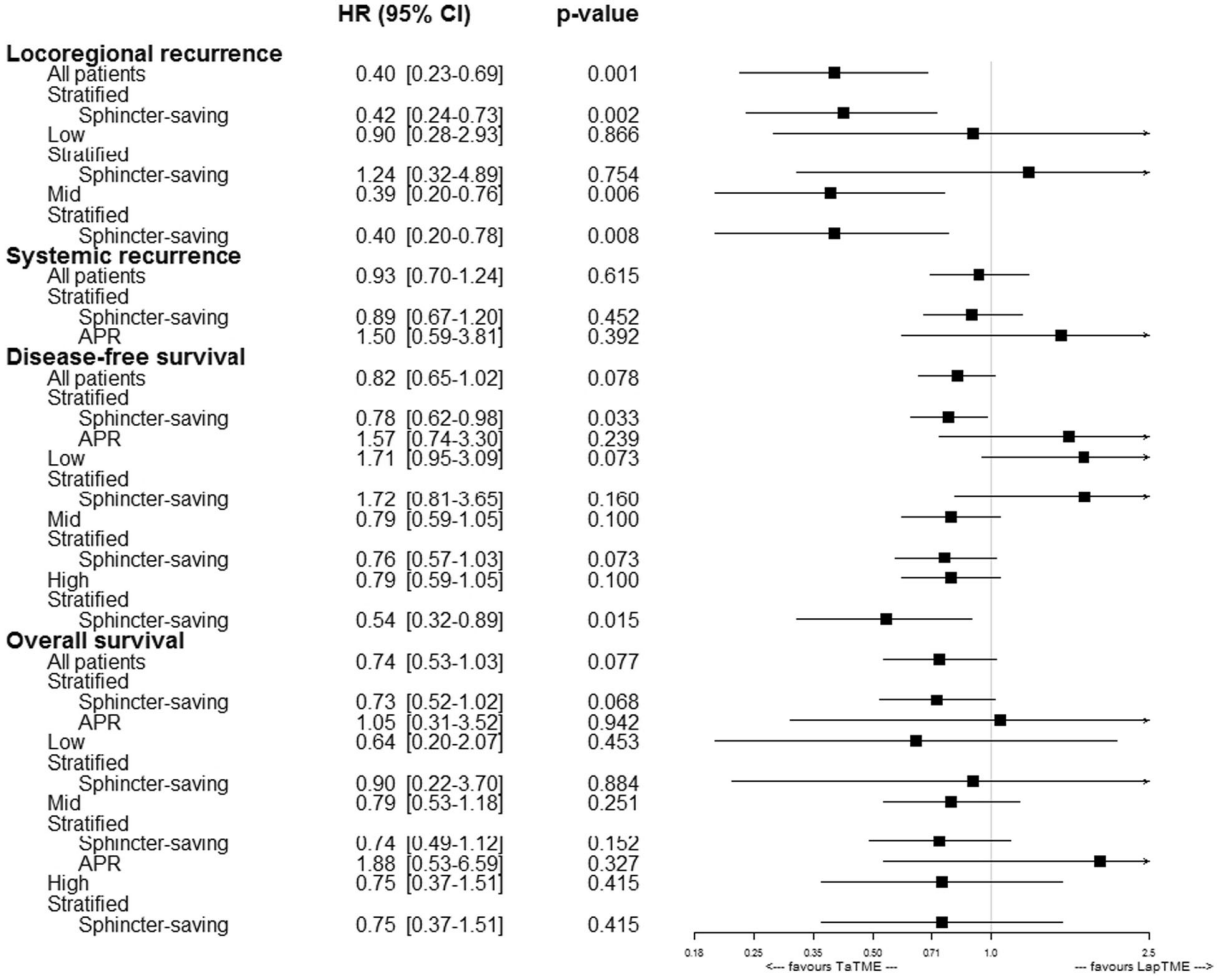
Subgroup analyses of survival and recurrence among patients with locally advanced rectal cancer treated with TaTME or LapTME. Abbreviations: HR, hazard ratio; CI, confidence interval; APR, abdominoperineal resection; TaTME, transanal total mesorectal excision; LapTME, laparoscopic total mesorectal excision

At 3 years, the disease-free survival rates were 74.3% in the TaTME group and 68.6% in the LapTME group HR = 0.81 (95% CI, 0.65–1.02; *p* = 0.078) (Fig. 3). However, when the analysis was limited to patients with sphincter preservation, an improved disease-free survival was observed in patients who underwent TaTME HR = 0.78 (95% CI, 0.62–0.98; *p* = 0.033). The overall survival rates were 87.2% in the TaTME group and 82.2% in the LapTME group HR = 0.74 (95% CI, 0.53–1.03; *p* = 0.076). Significant differences in overall survival could not be demonstrated in patients who underwent TaTME with sphincter preservation HR = 0.73 (95% CI, 0.52–1.02; *p* = 0.068). The survival and recurrence subgroup analyses are shown in Fig. 4.

Within 3 years after primary rectal cancer surgery, 35 locoregional recurrences were observed in the LapTME cohort, with a corresponding number of 12 in the TaTME cohort. Of those patients, 25 (six in the TaTME group and 19 in the LapTME group) presented with at least one of the following risks factors: T4 tumour, N2 disease, incomplete mesorectal specimen, or positive circumferential resection margin. The median time to locoregional recurrence could not be calculated since the event rate was less than 50%. However, the Accelerated Failure Time analysis identified a longer time-ratio in the TaTME group TR = 2.3 (CI, 1.34–4.00; *p* = 0.026). No multifocal pattern of recurrence was diagnosed.

## Discussion

In this multicenter cohort of 710 patients with clinical stage II-III rectal adenocarcinoma, TaTME provided a three-year 60% risk reduction for locoregional recurrence compared to LapTME. In patients undergoing surgery with sphincter preservation, the three-year disease-free survival rate was higher for patients treated with TaTME than for patients treated with LapTME. These benefits could be explained by an improved quality of the mesorectal specimen, with fewer positive resection margins, and lower rate of rectal perforations [4, 22, 23].

The performance of an optimal TME is technically demanding, and the histopathological equivalence of the laparoscopic and open approaches has been recently questioned [13, 24]. Fleshman et al. compared LapTME to open TME and included similar patients as the current study, except for the fact that every patient received neoadjuvant therapy. They found that LapTME did not meet the criteria for noninferiority in a composite score of complete or near-complete TME, and negative circumferential and distal resection margins [13]. It is important to note that Fleshman et al. did not find any difference in survival within 2 years after surgery [25]. Still, the study was not designed as an equivalence trial for survival and recurrence, and the absence of numerical distinctions might not be indicative of any dissimilarity.

Nevertheless, several clinical studies and meta-analyses have reported improved histopathological outcomes with TaTME compared to LapTME [26, 27]. However, the translation of these potentially advantageous data into improved mid- and long-term oncological outcomes is still scarce. The outcomes of the present study indicate that TaTME not only allows patients to benefit from the short-term advantages of minimally invasive surgery, but might also be superior in ensuring mid-term locoregional recurrence and disease-free survival in selected patients.

The reported improvement in locoregional recurrence rates was at the expense of patients with cancer of the mid rectum, but unexpectedly this was not confirmed in patients with cancer of the low rectum. This apparent disparity could be explained by a high proportion of patients, not estimable due to retrospective access to the data, in the LapTME group with low rectal tumours who underwent open perineal dissection using the transanal way to facilitate the most challenging part of the procedure. According to a randomised trial, transanal perineal dissection has been shown to decrease the risk of circumferential resection margin involvement by more than 4-fold compared to a purely abdominal TME [28]. Although further investigation is required, these combined data suggest that the surgical therapy of mid and low rectal cancer should include a perineal approach. This might be performed with conventional open surgical instruments or through TaTME in the low rectum. Given the difficulties of approaching mid rectal cancers using an open transanal technique (the Transanal Abdominal Transanal (TATA) procedure) and the inferiority of a pure transabdominal laparoscopic approach as suggested by the present data, TaTME might be the preferred technique when the tumour is in the mid rectum. Robot assisted transabdominal laparoscopy might potentially achieve similar results, but this has still to be confirmed.

Although knowledge in the literature is still scarce and follow-up periods are relatively short, the reported data that TaTME might be associated with a lower risk of locoregional recurrence are substantiated by several observational studies. Tuech et al. analysed 56 consecutive patients with low rectal cancer treated with TaTME and reported a locoregional recurrence rate of 1.7% with a median follow-up of 29 months [29]. Veltcamp Helbach et al. analysed 80 patients with mid- or low rectal cancer who underwent TaTME, and the locoregional recurrence rate after 2 years was 2.5% [16]. With a median follow-up of 31.9 months, Lelong et al. reported a 0% locoregional relapse rate [30]. More recently, Hol et al. analysed 159 consecutive patients undergoing TaTME with a complete and minimum follow-up of 3 years, reporting three- and five-year local relapse as low as 2 and 4%, respectively [31].

However, a recent study questioned the oncologic validity of the transanal approach. Larsen et al. reported, on behalf of the Norwegian Colorectal Cancer Group, that 9.5% of the 110 patients who underwent TaTME presented with an unexpected pattern of early locoregional recurrence, characterised by rapid, multifocal growth in the pelvic cavity and sidewalls [10]. The authors suggested that this atypical pattern of relapse may be a consequence of transanal pursestring failure, with spillage of the malignant cells that are aerosolised by the transanal insufflator. However, neither in the present study nor any of the published studies to date has revealed an unexpected pattern of recurrence. Moreover, the Accelerated Failure Time analysis of our study identified TaTME as a protective factor on patients’ locoregional recurrence time, suggesting a longer time to pelvic relapse in that group compared to patients treated with LapTME.

This clinical research study was based on real-world clinical practice and involved several groups of surgeons to enhance external validity. However, a significant limitation is its nonrandomised design. Observational studies are more susceptible to biases, even more so with surgical interventions where the risk of treatment assignment partiality is increased during the early phase of the learning curve. To avoid this allocation bias, we decided to use the inverse probability of treatment weight method, which has been shown to deliver results more comparable to an RCT than other techniques such as propensity score stratification and, unlike matching, retains most participant data [32]. After weighting, the covariates of the sample obtained were well-balanced and independent of treatment assignment. The only exception was the covariate type of surgery, which was applied as an adjustment cofactor in the Cox models. Nevertheless, we were unable to correct for unknown cofounders, and the analysis by subgroup depending on the type of surgery should be interpreted with caution due the low number of abdominoperineal resections in the TaTME cohort.

Another limitation is the inherent retrospective design which used historical controls. Besides, the extramural venous invasion variable could not be included in the propensity score model due to the large amount of missing data. This occurred predominantly in the LapTME group because the radiological extramural venous invasion has recently begun to be described. Variables such as budding, perineural and lymphovascular invasion displayed a heterogeneous distribution. However, the increased risk of recurrence that this may carry seems to be offset across the groups. Nonetheless, despite our extensive corrections, the presence of cofounders that might bias the results cannot be excluded. Finally, a representative non-selected group of patients who were treated for locally advanced rectal tumours was included. However, the surgical teams have extensive experience performing transanal procedures, and the results may not be generalised to other clinics that have recently started to perform TaTME.

## Conclusions

The results of this multicenter observational trial support a possible role for TaTME in improving locoregional recurrence and disease-free survival rates among patients with locally advanced rectal cancer. Further investigation in a randomised clinical trial is warranted.

## Supplementary information

**Additional file 1: Supplementary file 1. DF_AC** (ICMJE Form from Dr. Antoni Castells).

**Additional file 2: Supplementary file 2. DF_AML** (ICMJE Form from Dr. Antonio M. Lacy).

**Additional file 3: Supplementary file 3. DF_AO** (ICMJE Form from Dr. Ana Otero).

**Additional file 4: Supplementary file 4. DF_CS** (ICMJE Form from Dr. Colin Sietses).

**Additional file 5: Supplementary file 5. DF_FBL** (ICMJE Form from Dr. F. Borja de Lacy).

**Additional file 6: Supplementary file 6. DF_JR** (ICMJE Form from Mr. José Ríos).

**Additional file 7: Supplementary file 7. DF_JVL** (ICMJE Form from Dr. Jacqueline van Laarhovene).

**Additional file 8: Supplementary file 8. DF_PJT** (ICMJE Form from Dr. Pieter J. Tanis).

**Additional file 9: Supplementary file 9. DF_RB** (ICMJE Form from Dr. Raquel Bravo).

**Additional file 10: Supplementary file 10. DF_RH** (ICMJE Form from Dr. Roel Hompes).

**Additional file 11: Supplementary file 11. DF_RvP** (ICMJE Form from Dr. Roy van Poppel).

**Additional file 12: Supplementary file 12. DF_SV** (ICMJE Form from Dr. Silvia Valverde).

**Additional file 13: Supplementary file 13. DF_SXR** (ICMJE Form from Dr. Sapho X. Roodbeen).

**Additional file 14: Supplementary file 14. DF_TV** (ICMJE Form from Dr. Tjaakje Visser).

**Additional file 15: Supplementary file 15. DF_WAB** (ICMJE Form from Dr. Willem A. Bemelman).

## Data Availability

The datasets used and analysed, together with the generated syntaxes (coding), during the current study are available from the corresponding author and Mr. José Ríos on reasonable request.

## Abbreviations

TME: Total mesorectal excision
TaTME: Transanal total mesorectal excision
LapTME: Laparoscopic total mesorectal excision
MRI: Magnetic resonance imaging
ASA: American Society of Anaesthesiologists
M1: Metastatic tumours
TRG: Tumour regression grade
BMI: Body mass index
CI: Confidence interval
TR: Time ratio
HR: Hazard ratio
TATA: Transanal abdominal transanal procedure
